# Thermal Micro-Environment during Poultry Transportation in South Central United States

**DOI:** 10.3390/ani9010031

**Published:** 2019-01-21

**Authors:** Douglas J. Aldridge, Kaushik Luthra, Yi Liang, Karen Christensen, Susan E. Watkins, Colin G. Scanes

**Affiliations:** 1 Department of Poultry Science, University of Arkansas, Fayetteville, AR 72701, USA; djaldrid@uark.edu (D.J.A.); swatkin@uark.edu (S.E.W.); cscanes@uark.edu (C.G.S.); 2 Department of Biological & Agricultural Engineering, University of Arkansas, Fayetteville, AR 72701, USA; kluthra@uark.edu; 3 Tyson Foods, 2200 W Don Tyson Pkwy, Springdale, AR 72762, USA; Karen.Christensen@tyson.com

**Keywords:** broiler transport, thermal micro-environment, heat stress, animal welfare

## Abstract

**Simple Summary:**

This project monitored the internal micro-environments of live poultry transport trailers during loading and transport. For the 28 trips evaluated, trailers were modified using common USA industry mitigation practices designed to optimize bird comfort under a wide range of environmental conditions. In the cold season, double boarding of the exterior area of the transport modules elevated the internal temperature more than 8 °C above ambient temperatures as low as −16 °C. However, the temperature elevation may not be sufficient when ambient temperature was below 0 °C. In the warm season, surface wetting of birds and evaporative cooling applied during on-farm loading maintained trailer thermal conditions at or below ambient temperature for part of the road transport. However, this study suggests that additional improvement in equipment design or management is warranted when temperatures are extremely cold or hot.

**Abstract:**

This observational study was conducted to characterize the thermal micro- climate that broilers experienced in commercial poultry transporters under various weather conditions and typical management practices in the South Central USA. We continuously monitored temperature and relative humidity in 45 interior locations of 28 fully-loaded commercial trailers over 2 year spans from 2015–2016 in South Central USA. In the cold season, double boarding of the exterior area of the transport modules maintained temperatures at least 8 °C warmer than ambient temperatures as low as −16 °C. Overall, temperature at all locations decreased as transporters traveled from farms to processing plants during winter trips with double boards. In the hot season, assistance by evaporative cooling during on-farm loading resulted in interior temperatures within ± 2 °C of ambient conditions (up to 36 °C) during road transport. In the summer months, trailers uniformly gained 2 °C as vehicles travelled for 45 min from farms to plants. Apparent equivalent temperatures of the monitored summer trips averaged 80.5 °C, indicating possible heat stress conditions based on the thermal comfort zones defined by literature index values. For longer trips, cooling assistance on the farms may be insufficient to prevent temperatures from rising further into extremely hot conditions in the transporters, leading to a dangerous thermal environment.

## 1. Introduction

Market broilers face a variety of stresses, including feed and water withdrawal, vehicle vibration, and noise during live hauls from farms to processing plants. Among these, complex thermal environments have been identified as a major factor inducing physiological stress [1,2,3,4,5,6,7,8,9], with the most stressful stimuli being the extremes of heat and cold, contributing to seasonal-elevated “dead on arrivals” (DOAs). The welfare of the birds may be compromised either by a combination of air movement, low ambient temperatures (T) and winter precipitation that may cause cold stress, or the more prevalent problem of heat stress during transport [3].

Previous studies have attempted to characterize the behavioral and physiological responses of poultry in transportation in either field or lab conditions [1,6,7,8,10]. In a Canadian study, the transportation of three to four hours at temperatures below 0 °C was severe enough to decrease the internal body temperatures in broiler chickens [11,12]. Burlinguette et al. [8] reported a near-uniform temperature profile for trailers when ambient temperature was in the range of 8 to 11 °C operated in Saskatoon, Canada. However, when roll-up-style tarpaulin side curtains and most roof vents were closed at ambient temperature below −20 °C, temperature variation as large as 40 °C was observed at different modules in trailers. Ritz et al. [13] recorded black globe temperatures on 24 summer transport trailers in southeast United States, and reported that transport did not appear to exacerbate the temperatures experienced by broilers when the trailers were kept moving. 

Ideally, the exposure temperature of broilers should be within the thermoneutral zone of the birds, i.e., within the range of the conditions under which a bird can control its body temperature without altering its metabolic rate. Webster et al. [5] suggested a thermoneutral range for a well-feathered broiler from 8 to 18 °C under higher density in moving vehicles, lower than that of the birds in a typical rearing environment (24 to 28.5 °C) [14]. 

**Figure 1:**
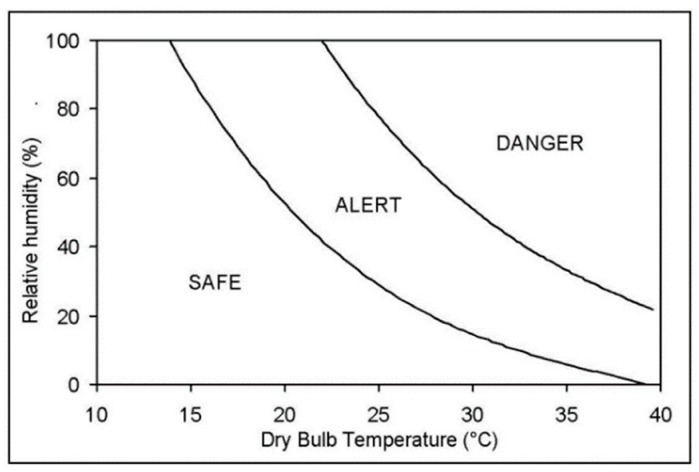
Thermal Comfort Zones for broiler transport defined by apparent equivalent temperature (AET) by Mitchell et al. [4]. Safe limit AET < 40 °C; danger limit Apparent equivalent temperature (AET) > 65 °C. Alert zone indicates moderate thermal stress with some degree of hyperthermia and acid-base disturbances; danger zone indicates severe thermal stress.

Quantifying heat loss of broilers on live-haul transport trailers is important to the understanding of the welfare of the broilers during this process [15], but is difficult to accomplish [16]. As an alternative, thermal comfort indices have been developed to assess the impact of the thermal environment on thermoregulatory status of animals. Mitchell et al. [4] developed the apparent equivalent temperature (AET) by incorporating the effect of temperature and humidity as an integrated index to correlate to changes of core body temperature under various T and humidity combinations (Figure 1).

The commercial vehicles used to transport broilers in South Central USA differ in their design from those used in Europe or Canada in terms of module materials, size, and weatherization measures. Trip lengths, bird market weight and seasonal stocking density may be different as well [5,8,10]. The objectives of the current study were to characterize the thermal micro-environments encountered by broilers in commercial poultry transporters under various weather conditions and typical management practices in the South Central USA.

## 2. Materials and Methods 

### 2.1. Trailer Description

**Figure 2:**
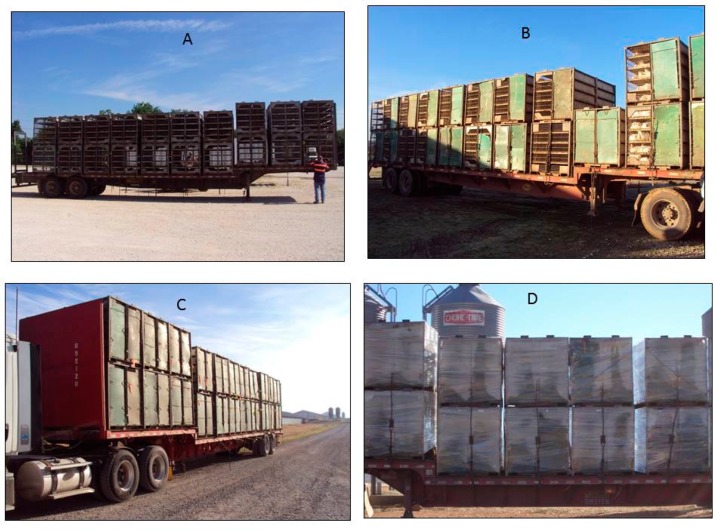
Weather-dependent trailer configurations employed by companies. **A**. Open, used for hot and mild seasons; **B.** Single board, used when transitioning between mild and winter seasons; **C**. Double board used in winter seasons; **D**. Plastic wrap on double board modules, used in winter days or nights with extremely cold conditions.

This study was carried out in Northwest Arkansas in collaboration with commercial companies during 2015 and 2016. In Southern USA, broilers are transported by galvanized steel modules (2.4 × 1.17 × 1.2 m) arranged on a flatbed trailer measuring 14.4 × 2.4 m (Table 1). Twenty-two modules are stacked in 11 rows on the trailer (Figure 2), with modules stacked in two tiers. Each module consists of a sheet metal roof and 10 perforated compartments arranged as five by two drawers (referred to 10-door cages). Each compartment had a solid plastic floor and a hinged solid door at the front of the compartment (1.2 × 0.21 m) for loading and unloading chickens. 

### 2.2. Trailer Setup by Industry Practices

During the road transport, broilers were exposed to passive ventilation. Company live haul personnel used different management practices to mitigate seasonal impact on broilers. In mild and hot seasons, trailer modules were completely exposed to the weather (“Open”, Figure 2A). In extremely cold conditions, fiberglass panels were screwed onto the exterior sides of the modules to reduce wind (called Wind board). The size of the wind board was designed to leave gaps on all edges of the module so that air can penetrate through the wires. Wind boards were installed progressively, first to cover approximately half of the exterior area of sides on each module in the fall season (refers to “Single board”, Figure 2B), followed by covering about 90% of the area during the winter months (“Double board”, Figure 2C). Once boards were on, they became permanent for the season. The boards were uninstalled in reverse fashion in the spring. Under extremely cold conditions or when precipitation coincided with low temperatures, some companies wrapped double-board modules using thin plastic film (70 gauge, 50.6 cm wide) immediately after birds were loaded into a module (“Plastic wrap”, Figure 2D). This treatment was not fixed to the modules like the boards, allowing the integrator to select specific loads to apply the wrap to. The wrap was applied horizontally, leaving the solid top and bottom of the module uncovered by the wrap. The module was individually wrapped inside the broiler house before it was moved onto the trailer by a fork-lift. Special care was taken during the loading of the modules to avoid any edges of adjacent modules touching that could result in cuts in the wrap. Wrapping all modules for a trailer required an additional 30 min. 

In the summer months, companies employed convective and evaporative cooling, i.e., fans and misting systems, to assist loading on farms during the daytime. In general, a fan assembly, consisting of a single or a linear array of multiple propeller fans spaced evenly, was positioned close to the transport trailer, and blows air into one side of it (“Cooling Assist”), while catching personnel brought modules with birds from a house and stacked them on the trailer from the opposite side. Water from misters on the fan assembly was blown toward the trailer under loading. Occasionally, a hand-held pressure washer attached to a house faucet was operated to apply water to the trailer during loading.

### 2.3. Data Collection of Live Haul Trips

The data were collected from six broiler processing plants of four companies from 2015 to 2016. Catching personnel loaded broilers into the modules. The majority of the cold-season monitoring trips were conducted at two processing plants slaughtering small-size broilers (nominal weight of 1.7 kg). Loading densities ranged from 34 to 36 birds per drawer (with floor space of 1.35 m^2^ per drawer), or 43 to 45 kg m^−2^. Warm-season monitoring trips were conducted at four processing plants slaughtering medium-size broilers (nominal weight of 3.0 kg). Loading densities ranged from 20 to 22 birds per drawer, or 44 to 49 kg m^−2^. Monitoring occurred during the daytime or nighttime in all seasons. The test procedures of this study were approved by the Institutional Animal Use and Care Committee of the University of Arkansas under Protocol # 15026.

Trailer boarding or cooling assistance were applied at the discretion of the live haul personnel. Thirty-three trips were monitored in total, with journey time ranging from 15 to 125 min (median of 60 min). The monitored trip lengths may not represent the typical journey lengths in this concentrated broiler production area, since we intentionally selected longer journeys to monitor due to the perceived potential challenges faced by broilers during long hauls. Journeys shorter than 40 min or with partial trailer loads were excluded from this report, leaving 28 trips for further analysis (Table 2). On two separate winter days, i.e., 11 January 2016 & 19 December 2016, two trailers either double board or plastic wrapped were instrumented identically and moved chickens from the same farm to the same plant at night; this allowed us to compare impact of wrapping on the thermal environment. 

### 2.4. Instrumentation

**Figure 3:**
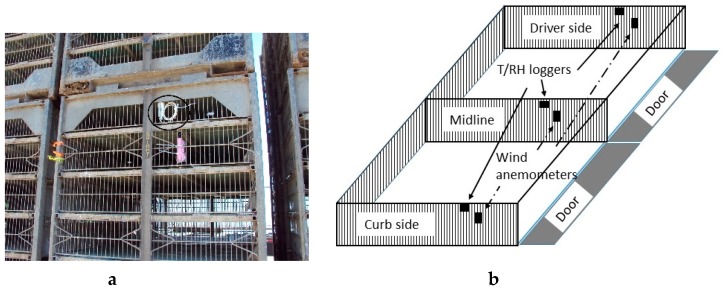
(**a**) Thermal and wind dataloggers secured on curbside of a transport module during a monitoring campaign. (**b**) Diagram of instrumented transport drawers showing loggers on curb side, midline and driver side. The interior loggers consist of those on the midline, while exterior loggers consist of those on driver and curb sides.

**Figure 4:**
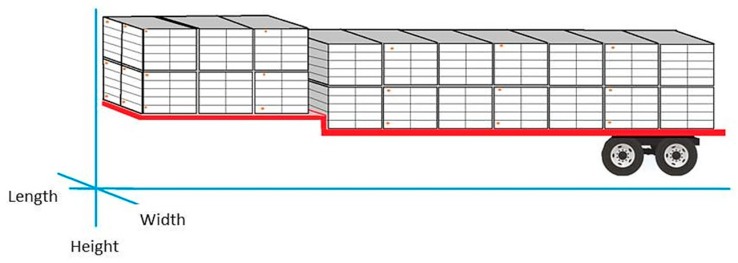
Schematic of an industry-standard drop deck poultry trailer loaded with 20, 10-door modules. Dataloggers (shown as orange dots) for temperature were installed as a 5 × 3 × 3 grid on the trailer. Three horizontal planes along height are Top, Middle, and Bottom. Three longitudinal planes along width are Driver, Midline and Curb.

Temperature and relative humidity (RH) (Hobo U23 Pro v2, −40 °C to 70 °C, ± 0.2 °C, and 0 to 100%, ± 2.5%, Onset Computer Corporation, Bourne, MA, USA) were recorded continuously at 25 locations throughout the trailer with T recorded at additional 20 locations (DS1921L Thermochron, ± 0.5 °C, Maxim Integrated, Sunnyvale, CA, USA). Air speed were measured by six anemometers (Kestrel 4000; accuracy ± 0.1 m s^−1^ from 0.4 to 40 m s^−1^, Minneapolis, MN, USA) on two modules. Before each scheduled monitoring campaign, data loggers were installed at pre-determined module locations on empty trailers using cable ties at the processing plant (Figure 3a,b). Nine thermal loggers occupied each of the five vertical cross-sections (Figure 4). Temperature, humidity and wind were measured every 30 s. Times of departure from farm to the processing plant were recorded using a GPS unit (eTrex 20, Garmin, Olathe, KS, USA) that logged waypoints every second, downloaded after each trip.

### 2.5. Data Analysis

Dry-bulb (T_db_), dew point temperatures (T_dp_) and relative humidity data were downloaded from the data loggers after each monitoring campaign. Ambient T and humidity corresponding to the monitored duration of each trip were obtained from weather data downloaded from nearby weather stations on the days that monitored trips took place.

Apparent equivalent temperature (AET) were calculated from the dry bulb temperature and the relative humidity [17]. Specifically,

(1) AET=T+10(30.5905−8.2×log10(K)−3142.31K)×(RH100)0.93×(0.0006363601×K+0.472)

where T = recorded air temperature, °C, K = T corrected to Kelvin (°C + 273.15), RH = recorded relative humidity, %.

Except for mean comparison of recorded temperature, elevated temperature above ambient values (i.e., Δt) were calculated by subtracting mean ambient temperature of each transit from the recorded temperature at each measurement location. For each management configuration, elevated temperature at three longitudinal planes (width, i.e., driver, midline and curb), at three horizontal planes (height, i.e., top, middle and bottom), and at five cross-section planes (Figure 3) were analyzed by ANOVA with means separated by Tukey’s range test [18].

Mean comparison of elevated temperature of different transit segments at three longitudinal and three horizontal planes of plastic wrap and double board were made [18]. The first segment was chosen as 15-min, due to an observed fast change immediately after the trailers departed from the farms, especially in summer months. Other segments were 30-min long. Only data from four segments were retained for this analysis. Differences of segment-average temperature were analyzed within groupings for each plane along the width and height axes [18], and considered significant if *p* < 0.05. Additionally, mean comparisons of elevated temperature above ambient (Δt) at different planes of the two paired trailers with either plastic wrapped or double board were made using paired t-test to determine the effect of plastic wrap on the double board winter trips. 

Relative humidity values represent how close air is to saturation at the measured temperature. Due to its temperature dependence, it is invalid to compute averages of recorded relative humidity over several hours directly. A “representative” relative humidity variable (RH*) was derived from a time-averaged humidity ratio (also called absolute humidity) and a corresponding time-averaged temperature from the same logging interval [19]. It serves as an “averaging” variable of relative humidity using appropriate psychometric manipulation.

Specifically, for each data logger, a humidity ratio (W) was computed at a specific time using its corresponding T and RH values [20]. After calculating the humidity ratio for a x time interval, a time-averaged humidity ratio ($\bar{W}$) and a time-average temperature ($\bar{T}$) were calculated for this duration. Before the representative RH* can be calculated, the partial pressure of water vapor in moist air (p_w_) was calculated using:

(2) $$p_{w}=\frac{\bar{W}\times p}{\left(\bar{W}+0.62198\right)}$$

where p_w_ = partial pressure of water vapor in moist air (Pa), $\bar{W}$ = time-averaged humidity ratio, p = total pressure of moist air assumed equal to atmospheric pressure (Pa).

The representative relative humidity was determined by:

(3) $${\mathrm{RH}}^{*}=\frac{P_{W}}{P_{\mathrm{WS}}}\times 100$$

where RH* = representative relative humidity (%), p_ws_ = saturation pressure of moist air (Pa). 

## 3. Results and Discussion

**Figure 5:**
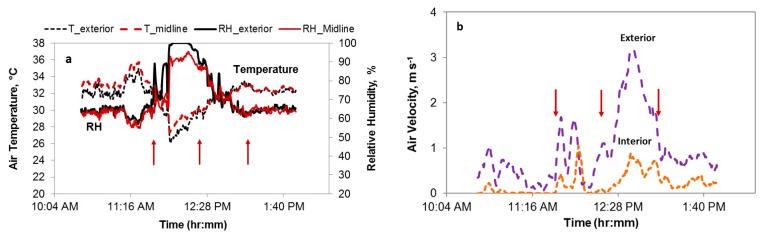
Temperature, relative humidity (**a**) and air velocity (**b**) profiles from interior and exterior logger positions of a summer trip. The first arrow on each graph indicates the beginning of loading, the second the beginning of transport, and the third the beginning of holding period.

Figure 5 illustrates examples of the temperature, RH and air speed profiles of interior and exterior of a summer live-haul trip. The ambient temperature at the start of this transport was 30.6 °C (Figure 5a). The first arrow indicates the beginning of loading, the second the beginning of transport, and the third the arrival at the processing plant for holding period. Cooling, including convective fans and water sprays, was applied to the trailer during loading, resulting in a sharp temperature drop and relative humidity increase. Air speeds during the 40-min transport were variable (Figure 5b), likely in part determined by the speed of the vehicle. Air speeds at interior and exterior locations averaged 0.5 and 1.9 m s^−1^, respectively. Webster et al. [5] reported mean air movement of open trucks in motion of 3.3 m s^−1^ (range 0.0 to 8.9 m s^−1^) of commercial broiler transporters in England. Weeks et al. [10] calculated that air speeds in moving vehicles varied from 0.9 to 2.4 m s^−1^ with maxima of 6.0 m s^−1^. 

**Figure 6:**
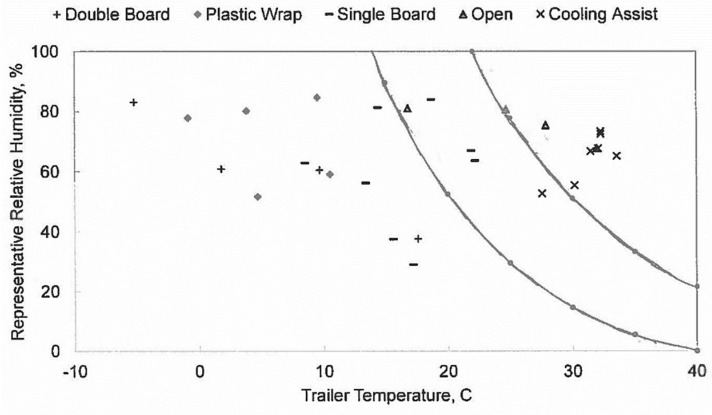
Average representative relative humidity vs. average trailer temperature of 28 monitored trips moving broilers to processing plants. The AET values corresponding to 40 and 65 °C [17] were overlaid.

The representative relative humidity of 28 trips and their trailer average temperatures are plotted in Figure 6. Using a physiological stress response model, Mitchell et al. [17] identified “safe”, “alert” and “danger” thermal zones, defined by AET values based on temperature-humidity combinations (Figure 1). AETs of 65 °C or greater were deemed dangerous due to potential severe physiological stress [17]. AETs of the monitored summer trips averaged 80.5 °C, indicating possible dangerous thermal conditions. Note that the laboratory experiments used to collect physiological parameters for derivation of the AET index were three hours in length with no air speed reported [17]. Majority of transport trips in this study area were less than two hours, with a median of one hour. Air speed on the moving trailers (Figure 5b) may have allowed convective cooling, although this was not uniformly experienced by all chickens on board. Additionally, partial surface wetting of broilers by hand sprayers may have alleviated or delayed the onset of heat stress of cooling-assisted transport trips based on literature reports [21,22]. 

The severity of physiological stress in the summer is unknown due to unavailable mortality data, which could have allowed correlation analysis with the thermal conditions. Future research should focus on improved research protocols, such as mortality data collection, measurements of core body temperature of broilers under various micro-environments, and behavior monitoring with video cameras. Nevertheless, it is important for commercial companies in South Central USA to improve the efficiency of the catching, loading and transporting process, and to minimize the duration of exposure of live chickens to uncontrolled environments in the summer. Additionally, better measures, such as stocking density adjustments, route optimization to avoid heavy traffic, and adding on-board sprinkler systems to the modules, should be considered for trips with longer distances to mitigate heat stress conditions.

Table 3 and Table 4 show the mean temperature and representative relative humidity that birds were exposed to for every 15 or 30-min transit duration of each trailer configuration. Under winter conditions, double boards and plastic wraps allowed heat produced by the broilers to be retained within the transporters, resulting in a mean trailer temperature of 5.5 °C (Table 3). While recorded trailer temperatures were higher than ambient T in winter (Table 3), they remained below the thermoneutral range [10], in spite of the boarding procedures to ameliorate the cold temperatures. Knezacek et al. [6] reported a 1 °C rectal temperature reduction of broilers that were exposed to 3.9 °C crate temperature during a 178-min winter trip of −28.2 °C ambient temperature. In a simulated 3-h wind tunnel experiment with chamber temperatures ranging between −4 and 12 °C, 1 °C rectal temperature reduction from broilers were reported under all dry chamber environments [23]. However, moderate to severe hypothermia (3 to 14 °C rectal temperature reduction) were observed when wetting was imposed in an increasingly colder chamber environment [23]. 

During the warm season with cooling assistance, overall trailer temperatures were within a narrow range of ambient conditions (up to 36.1 °C) for the duration of the journey (Table 3), with mean representative relative humidity less than 80% (Table 4). Both the air speed on trailers (Figure 5b) and the water retained by birds and trailer modules during on-farm loading could have prevented trailer air temperatures from rising far beyond ambient temperatures. Panting was observed from birds in transit based on camera footages from one selected summer trip, indicating that the efficacy of cooling assistance was limited due to a simultaneous increase of humidity level. 

Temperature throughout transporters with open and single side boards were mostly within the thermoneutral zone for broiler chickens (Table 3). Open transporters operating in the mild seasons provided a reasonably comfortable thermal environment. It is important to note that the greatest number of broilers are transported using the open sided configuration.

Humidity plays an important role in heat and mass exchanges in the livestock and poultry environment [4,15]. For example, moist air can compromise feather insulation properties, placing broilers at risk of cold stress [8,23]. Hunter et al. [23] concluded that broiler chickens could be safely transported at crate temperatures as low as −4 °C, if they are dry, or experience moderate hypothermia at temperatures as high as 8 °C when wet.

Representative relative humidity was selected to express the extent of moisture saturation in the modules. When moist air comes into contact with cooler surfaces (i.e., the modules and interior surface of wrapping plastics), condensation forms. Burlinguette et al. [8] used a threshold of 80% relative humidity value to determine susceptibility to condensation. In our study, mean representative relative humidity of four winter boarded transport was around 80% (Figure 6), indicating that a small amount of air exchange existed. Movement-induced ventilation prevented the excessive accumulation of moisture produced by the birds within the trailers (Table 4). 

### 3.1. Spatial Uniformity of Air Temperature on Trailer 

The industry practice of installing fiberglass boards on the modules is intended to reduce ventilation and conserve heat produced by the broilers in cool seasons. The practice seemed to be effective, elevating mean air temperature above their corresponding ambient temperature (with ranges of −15.8 to 2.8 °C and −16.4 to 8.9 °C, Table 2) by 10.7 °C and 9.3 °C for the plastic wrap and double board, respectively (Table 5). In comparison, three levels of curtains, and closure of roof vents used by Canadian transporters, resulted in an average temperature elevation of 14.4, 12.7 and 11.2 °C above ambient, as reported by Burlinguette et al. [8].

Differences in elevated temperature above ambient between locations were analyzed for each configuration. In winter, elevated temperatures at three longitudinal planes along the Width axis (Table 5) were different (*p* < 0.05) for all three boarding configurations. Mean elevated T at Midline were warmer than those at the outward-facing planes of the trailers (*p* < 0.05) when side boards were used, likely a result of lower airflow in the central locations. Top modules recorded mean T elevations of 8 °C from the ambient, which were several degrees lower than those gained by the middle or bottom modules, likely due to lack of protection from motion-induced ventilation. The lowest observed T was −1.1 °C at top, curb-side module, while the highest T, 18 °C, occurred on the midline, bottom module on double board trailers. This was similar to earlier report of highly variable and extreme thermal conditions when side curtains and most roof vents were closed on a Canadian transporter [8]. Large temperature gradients with up to 20 °C difference of crate temperatures (i.e., 3 to 26 °C) in an ambient temperature of −28 °C were reported when only the fourth roof vent was opened in a 178-min trip on a Saskatchewan transport trailer [6]. Kettlewell et al. [1] reported airflow movement from the back to the front of the trailers based on the temperature trends observed throughout trailers in the UK. However, the airflow distribution of the trailers in this study is unknown due to many undefined small openings at the back of each module (opposite to the door) (Figure 3b) and around the fiberglass boards. 

Transporters using cooling assistance displayed slightly different temperature profiles than those during cold or mild seasons. Temperatures tend to be higher (*p* < 0.05) at the midline (Table 5), although the difference was small (1.0 °C). The top tier displayed higher temperature elevations (*p* < 0.05), likely due to exposure of sheet metal roof to direct sunlight in summer. 

### 3.2. Effect of Journey Length on Thermal micro-Environment

Although journey lengths in this study were shorter (less than 2 h) compared to those reported elsewhere [1,6,10,19], elevated temperatures still differed from the beginning to the end (*p* < 0.05) (Table 6) for trips using plastic wrap. Trailer T elevation decreased significantly during transit at various locations in plastic wrap (up to 4.1 °C). A similar decline of elevated temperature above the ambient were observed in double board trailers without wrap (Table 7). 

When cooling assistance was used during loading in summer, temperatures increased from the first 15 min to the following 30 min across the trailer (*p* < 0.05) (Table 8). Air temperatures inside the trailers were lower than the corresponding outdoor conditions during the first 15 min immediately after trailers departed from farms. This was clearly the residual effect of liquid water retained on transporters from loading on farms. Fans and various types of water treatments, including misters and hand-held sprayers, were used in all eight trips monitored and reported in this category. Water retained by modules and birds’ feathers continued evaporating as transporters traveled on the roads. Ritz et al. [13] also reported that the use of multiple high-velocity fans positioned parallel to the live-haul trailers during loading was effective at cooling birds prior to transport. However, water likely diminished around 15 min after transporters’ departure, allowing temperature rises of 2 to 3 °C at various locations after one hour (*p* < 0.05, Table 8). For hot weather conditions, even with 1 to 2 °C temperature rises within the trailer, thermal load could shift to a more dangerous level. 

### 3.3. Effect of Plastic Wrappping on the Micro-Environment

Plastic wrap, in addition to the double boarded trailers, raised the mean air temperature by around 3.2 °C compared to double boarded trailers on winter nights with average ambient temperatures of −5 and −17 °C (Table 9). Average representative relative humidities of double board and plastic wrapped trailers were 72% and 79%, respectively (Figure 6). Plastic wrapping was only used to further reduce wind penetration through modules in order to protect birds from extremely cold weather conditions when birds with incomplete feather coverage (with 1.7 kg live weight) were transported. This practice seemed to moderately retain heat and water vapor inside the modules without creating moisture saturation. Better protection, such as more insulation, might be needed in order to alleviate cold stress without any risk of creating saturated air conditions.

## 4. Conclusions

Temperature and relative humidity were monitored in 45 locations on 28 commercial trips hauling market-size broilers to processing plants. Weather-dependent management employed by companies, including side boards attached on the open area of modules in winter and fan trailers with mists used during loading in summer, were analyzed for their effect on altering micro-environment of the trailers. During cold weather transport when ambient temperatures were below 0 °C, on-board temperatures were lower near the exterior than in the middle, and decreased steadily as transport duration increased. Trailer temperatures on double board trailers in winter averaged 8 °C above ambient T. During warm weather transport, on-board temperatures were within ± 2 °C of the ambient, and higher near the top module of the trailers. Temperatures throughout the trailer increased by 1 to 3 °C as transit time increased in summer. Apparent equivalent temperatures of the monitored summer trips averaged 80.5 °C, indicating possible heat stress conditions based on literature reported index values. Improvement in equipment or transport management would therefore be necessary during extremely cold or hot weather.

## Figures and Tables

**Table 1:** Dimensions (m) of the commercial broiler transporters, modules and drawers used in this study.

Component	Length	Width	Height
Trailer	14.4	2.4	2.5 ^1^
Module	2.4	1.17	1.2
Drawer	1.2	1.17	0.24

^1^ The height represents the stacked transport modules on open-bed trailers, which have no roof.

**Table 2:** Range of ambient temperatures of trailer configuration for 28 trips and their transit duration.

Ambient Temperature (°C)	Trailer Configuration		No. of Trips	Transit Duration, min
	Boarding	Cooling Assist at Loading		
−15.8 to 2.8	Plastic wrapped		5	75 to 125
−16.4 to 8.9	90% boarded		4	78 to 107
2.3 to 20.0	Half boarded		7	41 to 84
12.2 to 29.4			4	63 to 105
29.3 to 36.1		Fan + water	8	42 to 74

**Table 3:** Mean recorded temperature (°C) of 15 or 30-min segments during transit under five trailer configurations found in commercial broiler transporters.

Duration	Plastic Wrap	Double Board	Single Board	Open	Cooling Assist
Ambient	−5.3 ± 3.2	−3. 5± 5.4	10.0 ± 1.7	22.0 ± 6.3	31.5 ± 3.0
Transit:					
0–15 min	6.9 ± 2.6	6.6 ± 2.9	16.9 ± 2.1	25.0 ± 5.4	29.6 ± 2.2
15–45 min	6.2 ± 2.7	6.2 ± 3.1	16.6 ± 2.2	25.3 ± 5.6	32.1 ± 1.9
45–75 min	5.1 ± 3.2	5.8 ± 3.6	18.5 ± 3.5	25.2 ± 6.8	33.1 ± 1.1
75–05 min	3.7 ± 6.0	6.1 ± 5.7			

**Table 4:** Mean representative relative humidity (RH*, %) every 15 or 30-min during transit under five trailer configurations found in commercial broiler transporters.

Duration	Plastic Wrap	Double Board	Single Board	Open	Cooling Assist
Ambient	68.1 ± 6.8	71.5 ± 4.3	62.4 ± 10.0	82.4 ± 5.4	51.8 ± 4.2
Transit					
0–15 min	78.7 ± 2.8	70.6 ± 8.5	55.4 ± 7.4	76.7 ± 2.4	72.5 ± 4.3
15–45 min	74.7 ± 3.6	68.5 ± 6.7	56.3 ± 8.8	66.9 ± 1.8	70.4 ± 4.7
45–75 min	73.7 ± 4.6	67.6 ± 7.1	35.1 ± 8.8	64.2 ± 0.0	70.8 ± 6.3
75–105 min	74.5 ± 10.4	56.7 ± 9.3			

**Table 5:** Spatial variation of air temperatures elevation above-ambient (Δt, °C) across the trailer during transport in different seasons.

Location	Plastic Wrap	Double Board	Single Board	Open	Cooling Assist
Width					
Driver	9.9 ± 1.3 ^b^	8.1 ± 0.6 ^b^	4.8 ± 0.8 ^c^	4.1 ± 1.5 ^c^	−0.10 ± 1.0 ^c^
Midline	11.9 ± 1.3 ^a^	11.5 ± 0.6 ^a^	6.5 ± 0.8 ^a^	5.2 ± 1.5 ^a^	0.78 ± 1.0 ^a^
Curb	10.4 ± 1.3 ^b^	8.4 ± 0.6 ^b^	5.5 ± 0.8 ^b^	4.6 ± 1.5 ^b^	0.35± 1.0 ^b^
Height					
Top	8.7 ± 2.4 ^c^	8.2 ± 1.2 ^b^	5.4 ± 1.4 ^a^	4.6 ± 2.7 ^b^	1.7 ± 1.0 ^a^
Middle	13.1 ± 2.4 ^a^	9.8 ± 1.2 ^a^	5.6 ± 1.4 ^a^	4.5 ± 2.7 ^ab^	−0.20 ± 1.0 ^b^
Bottom	10.4 ± 2.4 ^b^	10.1 ± 1.2 ^a^	5.8 ± 1.4 ^a^	4.8 ± 2.7 ^a^	−0.03 ± 1.0 ^b^
Length ^1^					
1	10.0 ± 1.4 ^bc^	10.5 ± 0.7 ^a^	6.3 ± 0.8 ^a^	5.3 ± 1.5 ^a^	0.54 ± 1.0 ^a^
2	10.6 ± 1.4 ^b^	8.5 ± 0.7 ^c^	4.7 ± 0.8 ^b^	4.5 ± 1.5 ^b^	0.46 ± 1.0 ^ab^
3	13.5 ± 1.4 ^a^	10.4 ± 0.7 ^a^	6.5 ± 0.8 ^a^	4.9 ± 1.5 ^c^	0.46 ± 1.0 ^ab^
4	9.8 ± 1.4 ^bc^	9.4 ± 0.7 ^b^	5.2 ± 0.8 ^b^	4.4 ± 1.5 ^c^	0.18 ± 1.0 ^ab^
5	9.7 ± 1.4 ^c^	8.0 ± 0.7 ^c^	5.2 ± 0.8 ^b^	4.0 ± 1.5 ^d^	0.08 ± 1.0 ^b^

^a,b,c^ Superscripts denote differences (*p* < 0.05) within each column and axis, ^1^ Code 1, 2, 3, 4 and 5 denote instrumented cross sections from the front to the back of trailers.

**Table 6:** The effect of trip duration on elevated temperature above ambient (Δt, °C) at measured locations across width and height of the trailer with plastic wrap (*n* = 5).

Duration	Width			Height		
	Driver	Midline	Curb	Top	Middle	Bottom
0–15 min	11.3 ^a^	13.8 ^a^	11.7 ^a^	10.9 ^a^	14.7 ^a^	11.1 ^a^
15–45 min	10.6 ^ab^	12.6 ^a^	11.2 ^ab^	9.2 ^b^	14.1 ^a^	11.2 ^a^
45–75 min	9.8 ^b^	11.5 ^b^	10.2 ^bc^	8.3 ^b^	12.7 ^b^	10.5 ^a^
75–105 min	8.0 ^c^	9.7 ^c^	8.6 ^d^	6.6 ^c^	10.9 ^c^	8.7 ^b^

^a,b,c,d^ Superscripts denote differences (*p* < 0.05) within each column and axis.

**Table 7:** Effect of trip duration on elevated temperature above ambient (Δt, °C) at measured locations across the width and height of the trailer with double boards (*n* = 4).

Duration	Width			Height		
	Driver	Midline	Curb	Top	Middle	Bottom
0–15 min	10.1 ^a^	12.6 ^a^	10.0 ^a^	9.6 ^a^	11.5 ^a^	11.6 ^a^
15–45 min	8.8 ^b^	12.7 ^a^	8.9 ^ab^	8.8 ^a^	10.8 ^a^	10.8 ^ab^
45–75 min	7.8 ^c^	11.5 ^b^	8.2 ^bc^	7.9 ^b^	9.6 ^b^	10.0 ^bc^
75–105 min	5.9 ^d^	9.0 ^c^	6.7 ^d^	6.3 ^c^	7.3 ^c^	8.0 ^d^

^a,b,c,d^ Superscripts denote differences (*p* < 0.05) within each column and axis.

**Table 8:** Effect of trip duration on elevated temperature above ambient (Δt, °C) at measured locations across width and height of the trailer when cooling assistance was used (*n* = 8). Cooling assistance consisted of propeller fan(s) blowing air and misters or hand-held pressure washers applying water toward trailers being loaded.

Duration	Width			Height		
	Driver	Midline	Curb	Top	Middle	Bottom
0–15 min	−2.2 ^b^	−0.3 ^b^	−1.6 ^b^	−0.1 ^c^	−2.0 ^b^	−2.0 ^b^
15–45 min	0.8 ^a^	1.4 ^a^	1.3 ^a^	2.4 ^a^	0.4 ^a^	0.7 ^a^
45–75 min	1.2 ^a^	1.2 ^a^	1.4 ^a^	1.5 ^b^	1.0 ^a^	1.2 ^a^

^a,b,c^ Superscripts denote differences (*p* < 0.05) within each column of width and height axis.

**Table 9:** Means and standard errors of elevated temperatures above ambient (Δt, °C and the paired sample t-test at various locations of paired trailers with either plastic wrap or double board on two winter nights with average ambient temperatures of −5 and −17 °C, respectively.

Treatment	Width			Height		
	Driver	Midline	Curb	Top	Middle	Bottom
Plastic wrap	12.3 ^a^	14.6 ^a^	11.7 ^a^	10.5 ^a^	16.0 ^a^	11.9 ^a^
Double board	8.6 ^b^	11.8 ^b^	8.4 ^b^	8.0 ^b^	10.8 ^b^	10.3 ^b^
Stderr	0.10	0.12	0.12	0.06	0.13	0.11
t-value	40.0	24.3	27.3	14.7	39.5	39.4

^a,b^ Superscripts denote differences (*p* < 0.05) within each column.
